# Acceptance Commitment Therapy Intervention for Custodial Grandfamilies: A Virtual Implementation

**DOI:** 10.1093/geroni/igab046.3035

**Published:** 2021-12-17

**Authors:** Ella Faulhaber, Meghan Custis, Emily Heupel, Jeongeun Lee

**Affiliations:** 1 Iowa State University, Ames, Iowa, United States; 2 Iowa State University, MUSCATINE, Iowa, United States

## Abstract

Custodial grandfamilies often face challenges, such as psychological distress, parenting burden, and grandchildren showing internalizing and externalizing behaviors. Given their circumstances, effective training and education is critical to provide a supportive family environment for both custodial grandparents and custodial grandchildren. The aim of this study was to develop, pilot and evaluate a psychosocial intervention for custodial grandfamilies. The program, interACT, was implemented virtually due to restrictions related to COVID-19. It is an intervention for custodial grandparents (CGPs) and custodial grandchildren (CGC) to improve psychological wellbeing and life skills. Participant eligibility was determined by grandfamilies having Iowa residency and legal guardianship or custody of an 8-12 year old grandchild. The program utilizes the Acceptance and Commitment Therapy (ACT) framework, focusing on psychological flexibility, acceptance, and psychosocial resilience for CGPs and CGC. The program used techniques such as mindfulness, self-compassion, and decision-making skills. During the pilot program stage, participants were divided into CGPs and CGC groups, and completed self-paced weekly 45 minute long modules through a program website. To enhance online session experience, Extension staff facilitated hour-long Zoom calls to discuss module contents and foster peer connection for both groups. Findings will be available at the conclusion of the pilot program. We expect to find increased psychological well-being and improved life skills for both groups right after the implementation of the ACT. Findings and limitations will be discussed with practical implication for program implementation via virtual delivery for the current custodial families. Future studies could extend current curriculum to other populations.

